# Exercise Evaluation and Prescription in Older Adults

**DOI:** 10.3390/healthcare11010042

**Published:** 2022-12-23

**Authors:** Guilherme Eustáquio Furtado, Rubens Vinícius Letieri, Eduardo Carballeira

**Affiliations:** 1 Polytechnic Institute of Coimbra, Applied Research Institute, Rua da Misericórdia, Lagar dos Cortiços-S. Martinho do Bispo, 3045-093 Coimbra, Portugal; 2 Research Unit for Sport and Physical Activity (CIDAF, UID/PTD/04213/2020), Faculty of Sport Sciences and Physical Education (FCDEF-UC), Pavilhão 3, 3040-248 Coimbra, Portugal; 3 Physical Education Department, Multidisciplinary Research Nucleus in Physical Education (NIMEF), Federal University of Northern of Tocantins (UFNT), Avenida Nossa Senhora de Fátima, 1558, Centro, Tocantinópolis 77900-000, Brazil; 4 Department of Physical Education and Sport, Faculty of Sports Science and Physical Education, University of A Coruña, 15001 A Coruña, Spain

The over-65 age group is growing faster than other age groups worldwide [1]. Typically, older people are known to be less active than the younger population and are at greater risk for impaired overall health [2]. Within aging, it is essential to know the several factors that contribute to the overall well-being of older individuals. From this perspective, understanding aging from a multifactorial point of view is essential, and analysis of psychological, social, environmental, and functional factors helps to direct health promotion programs for this population. 

The impacts of social isolation due to the COVID-19 pandemic have driven the need for more careful observations about these factors. Intrinsic and extrinsic factors strongly contribute to the maintenance of health. However, there is an increasing recognition of the effects of health determinants throughout life, from birth to old age. In this process, it is evident that understanding social isolation and the factors that affect the quality of life of the elderly, including mental capacity, metabolic parameters, fear of falling, functional balance, and frailty, is extremely relevant [3]. From this point of view, Cezário and colleagues presented significant results by highlighting the importance of keeping an active lifestyle during the COVID-19 pandemic era aiming to maintain physical-functional and metabolic characteristics, in a study involving community-dwelling older people [4].

The impact of physical exercise performed in aquatic and land environments represents a set of medium- and long-term intervention studies in this Special Issue that deserve to be highlighted. Firstly, Martínez-Rodríguez and colleagues showed that when community-dwelling older people were involved in aquatic exercise programs, they perceived greater well-being and satisfaction because they met their basic psychological needs and autonomy in the face of the pressure of demands imposed by everyday life. As a result, participants reported heightened well-being and increased exercise adherence [5]. In another study, Rodrigues and collaborators demonstrated that strength exercise programs with accessible and cheap material (elastic bands) improved postural control and reduced both fall risk and fear of falling in older people with poor functional fitness status [6]. In addition, Mulasso and their colleagues demonstrated that a multicomponent exercise program (MEP) might improve and reduce frailty, confirming findings reported in previous studies involving MEP [7,8]. Lastly, the study conducted by Ramuth et al. showed that a weight-loss innovative method based on a ketogenic diet plan and associated with a fitness health coach’s assistance promoted improvements in health as well as safe, fast weight loss [9].

In the cross-sectional studies, Huang et al. [10], Nieto-Guisado et al. [11], and Souza et al. [12] demonstrated the close relationship between physical function, fear/risk of falling, proprioception, and postural control, reinforcing the importance of this type of screen as a means of preventing adverse outcomes associated with falls. Additionally, Santos et al. demonstrated the effectiveness of the jump test in assessing muscle strength and power [13], two main factors in developing older adults’ physical autonomy. Loss of type II muscle fibers is a primary negative outcome related to health status with aging; thus, the relationship between these losses and the high incidence of falls deserves special mention. An interesting perspective is emphasized by Furtado and his colleagues, which evaluated different components of physical fitness and quality of life in older people living in rural and urban environments [14]. These findings revealed that rural residents scored higher on quality of life and functional fitness indicators than urban residents, establishing a precedent for future research into the relationship between the health and well-being of older people living in these two types of environments.

Despite the comprehensive utility of physical exercise [15] and the heterogeneity responsiveness to regular exercise, geroscience needs further studies of exercise and health using individualized responses [16,17,18]. Researchers are encouraged to design studies to identify effective methods of increasing engagement and maintaining adherence to training in older adults. Furthermore, it is essential to evaluate older adults’ responses to training at the individual and group levels and identify sources of variability in responsiveness to training [19]. Specifically, aging is a very heterogenous process where genetics and life habits entail different health statuses. Thus, we must enhance our knowledge of strategies for optimizing exercise dosing to maximize benefits while minimizing barriers to participation and the efficacy of multimodal interventions for relevant subgroups of older adults. Future research on this topic is needed to evolve exercise prescription and evaluation into a personalized form of medicine.
